# Primary Hepatoid Adenocarcinoma of the Lung With Signet Ring Cell Differentiation and Widespread Osseous Metastases: A Case Report

**DOI:** 10.1002/rcr2.70604

**Published:** 2026-05-06

**Authors:** Rami Khader, Esraa Elhakim, Mohammad Bdair, Rajaa Abu Sultan, Ameed Khader, Mousa Almasalma, Saleh Ferwana, Mohammed Z. A. Baraka

**Affiliations:** ^1^ Faculty of Medicine Mansoura University Mansoura Egypt; ^2^ Medical Oncology Unit, Oncology Center Mansoura University Mansoura Egypt; ^3^ Department of Medicine, Faculty of Medicine and Health Sciences An‐Najah National University Nablus Palestine; ^4^ Department of Medicine Alexandria University Alexandria Egypt

**Keywords:** alpha‐fetoprotein, bone metastasis, CK7, HepPar‐1, pulmonary hepatoid adenocarcinoma, signet ring cell

## Abstract

Pulmonary hepatoid adenocarcinoma (HAL) is an extremely rare and highly aggressive extrahepatic adenocarcinoma that exhibits hepatocellular carcinoma–like morphology and hepatocytic immunophenotype, often leading to diagnostic delay due to nonspecific respiratory presentations. We report a male patient in his 50s with a long history of heavy tobacco use who presented with a persistent cough initially treated as pneumonia without clinical improvement. Over subsequent weeks, he developed progressive dyspnea, weight loss, fatigue and diffuse bone pain. Computed tomography demonstrated left upper‐lobe consolidation with pleural effusion and underlying collapse, mediastinal lymphadenopathy, and multiple lytic skeletal lesions consistent with metastatic disease, while triphasic abdominal imaging showed no focal hepatic lesions. Laboratory testing revealed mild anaemia with leukocytosis and thrombocytosis, markedly elevated carcinoembryonic antigen and normal alpha‐fetoprotein. Tru‐cut biopsy showed poorly differentiated adenocarcinoma with prominent signet ring cell differentiation. Immunohistochemistry demonstrated diffuse CK7 and HepPar‐1 positivity with focal CDX2 positivity and negativity for TTF‐1, CK20, p63, and D2‐40, supporting a diagnosis of primary pulmonary hepatoid adenocarcinoma. The patient received 3 cycles of gemcitabine plus carboplatin but experienced rapid clinical deterioration. This case underscores that HAL may mimic nonresolving pneumonia and that normal AFP does not exclude the diagnosis; early biopsy and a targeted immunohistochemical panel are essential for timely recognition of this lethal entity.

## Graphical abstract

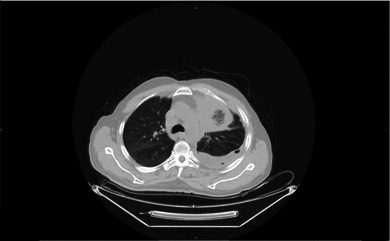

## 1 Introduction

Hepatoid adenocarcinoma is an uncommon and aggressive variant of adenocarcinoma with morphologic and immunophenotypic features similar to hepatocellular carcinoma (HCC), but it arises in organs other than the liver [1]. Hepatoid adenocarcinoma was first described in gastric cancer, and it has been observed in various organs, such as the ovary, pancreas, colon, urinary bladder and lung. Of these, primary hepatoid adenocarcinoma of the lung is extremely rare, and few cases have been reported in the literature [2, 3].

Pulmonary hepatoid adenocarcinoma mainly occurs in middle‐aged and old men and is closely correlated to smoking history. Clinically, it commonly manifests as nonspecific respiratory symptoms including cough, dyspnea or chest pain and may be initially misdiagnosed as infectious or inflammatory lung disease [3, 4]. At presentation, many of the patients have already advanced disease with lymph nodal or distant metastasis, often to bone, liver and adrenal gland which are the most frequent sites of this tumour's aggressive biological behaviour [5].

Histology shows poorly differentiated adenocarcinoma with hepatoid features (polygonal tumour cells widely spread eosinophilic cytoplasm and sometimes presenting signet ring cell component). Immunohistochemical analysis is critical for making the correct diagnosis, especially for distinguishing lung hepatoid adenocarcinoma from pulmonary metastatic hepatocellular carcinoma [3]. The tumours express cytokeratin 7 and hepato‐cyte‐specific markers (e.g., HepPar‐1) while not expressing thyroid transcription factor‐1 and cytokeratin 20, which is consistent with a primary pulmonary site. Serum alpha‐fetoprotein can be raised in some cases but is not always elevated, and normal levels do not rule out a diagnosis [2].

Because it is a rare type of tumour, there is no consensus on the treatment for pulmonary hepatoid adenocarcinoma. Management of disease is typically derived from traditional non–small cell lung cancer protocols, often with platinum‐based chemotherapy with limited reported success. The prognosis is poor: most patients present with a rapidly progressive disease, which responds only marginally to treatment [3].

Herein we present a case of hepatoid adenocarcinoma in the lung which was initially misdiagnosed as pneumonia and rapidly progressed to widespread metastatic disease. This case emphasises the diagnostic difficulty, radiological and histopathologic characteristics, and aggressive clinical behaviour of this rare tumour and underscores the need for early recognition and thorough pathologic inspection.

## 2 Case Report

A male patient in his 50s with a significant history of heavy tobacco use presented in February 2025 with a persistent cough. He initially sought medical care outside our institution, where he was diagnosed with pneumonia and treated with antibiotic therapy. Despite treatment, his symptoms failed to improve and progressively worsened, with the development of dyspnea. Over the following months, he experienced unintentional weight loss, generalised fatigue and diffuse bone pain. Owing to the persistence and progression of symptoms, he presented to our medical oncology clinic in April 2025 for further evaluation.

The patient initially sought medical care at a chest clinic outside our institution. A chest radiograph demonstrated mild left‐sided pleural effusion with scattered non‐homogeneous opacities in the left mid‐lung zone. A diagnostic pleural aspiration was performed by a pulmonologist, and pleural fluid analysis was conducted at an external facility. Cytological examination revealed predominantly inflammatory cells with a white blood cell count exceeding 100 cells/μL, and microbiological cultures showed no bacterial growth. Histopathological assessment was reported as inflammatory. However, due to persistent symptoms and radiological suspicion of malignancy, the patient was referred to our oncology centre for further evaluation.

The patient's past medical history was notable for type 2 diabetes mellitus managed with oral hypoglycemic agents, hypertension, and coronary artery disease, for which he had undergone percutaneous coronary intervention with stent placement 1.5 years prior to presentation. He was maintained on clopidogrel therapy at the time of evaluation.

Initial laboratory investigations demonstrated mild anaemia, leukocytosis and thrombocytosis. Liver and renal function tests were within normal limits, as were coagulation parameters. Viral screening for hepatitis B virus, hepatitis C virus, and human immunodeficiency virus was negative. Tumour marker assessment revealed a markedly elevated carcinoembryonic antigen (CEA) level, while alpha‐fetoprotein (AFP) was within the normal range. A summary of laboratory findings is provided in Table 1.

**TABLE 1:** Laboratory investigations at presentation.

Parameter	Result	Reference range
White blood cell count	10.16 × 10^3^/μL	4.0–11.0 × 10^3^/μL
Haemoglobin	11 g/dL	13.0–17.0 g/dL
Platelet count	509 × 10^3^/μL	150–450 × 10^3^/μL
SGOT (AST)	20 U/L	< 40 U/L
SGPT (ALT)	38 U/L	< 41 U/L
Total bilirubin	0.9 mg/dL	0.2–1.2 mg/dL
Serum creatinine	0.9 mg/dL	0.6–1.3 mg/dL
Prothrombin time	13.1 s	11–14 s
INR	1.1	0.9–1.2
HBsAg	Negative	—
Anti‐HCV	Negative	—
HIV	Negative	—
CEA	94.4 ng/mL	< 5.0 ng/mL
AFP	1.15 IU/mL	< 10 IU/mL

**FIGURE 1:**
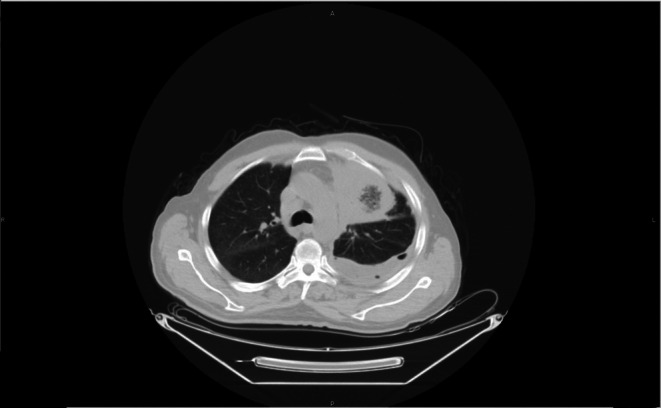
Non‐contrast CT chest showing left upper lobe consolidation with pleural effusion and underlying lung collapse.

**FIGURE 2:**
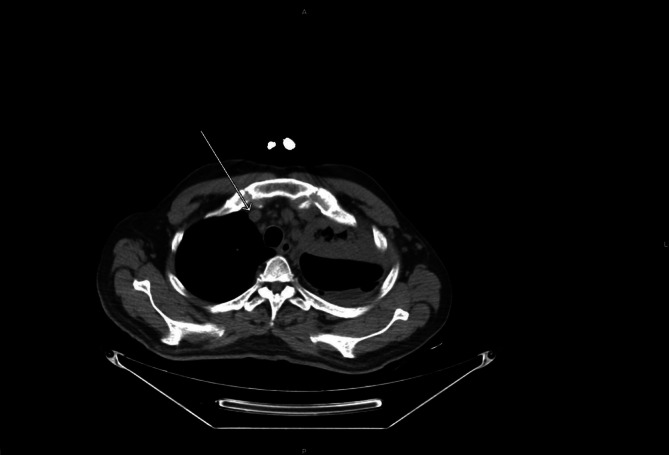
Mediastinal lymphadenopathy with enlargement of the right paratracheal lymph node.

A non‐contrast multi‐slice CT scan of the brain, neck, chest, abdomen, and pelvis was performed. The brain and neck showed no intra‐ or extra‐axial space‐occupying lesions, and no significant cervical or axillary lymphadenopathy was identified. Thoracic imaging revealed consolidation of the left upper lobe associated with mild to moderate left‐sided pleural effusion and underlying lower lung collapse (Figure 1). Small air foci were noted within the pleural space following diagnostic pleural aspiration. The right lung field was clear with no pleural effusion. Multiple enlarged mediastinal lymph nodes were identified, with the largest measuring 12 mm in short‐axis diameter in the right paratracheal region (Figure 2).

**FIGURE 3:**
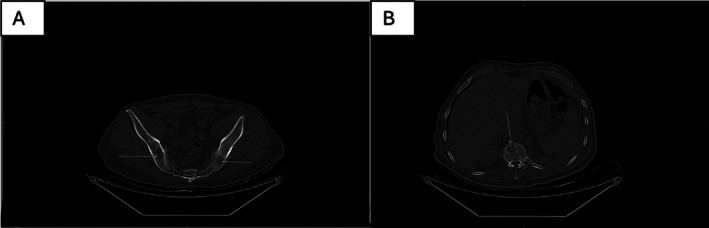
Non‐contrast axial CT images demonstrating multiple lytic lesions involving the pelvic bones and vertebral bodies: (A) bilateral iliac bone lytic lesions and (B) vertebral body lytic lesion, consistent with metastatic disease.

Skeletal imaging demonstrated multiple lytic lesions involving multilevel vertebrae and pelvic bones, highly suggestive of metastatic disease (Figure 3). The liver and spleen were normal in size and homogeneous in texture. Both kidneys were normal in size and parenchymal density, with no evidence of calculi or obstructive uropathy. The right suprarenal gland appeared bulky. No ascites or intra‐abdominal collections were detected.

**FIGURE 4:**
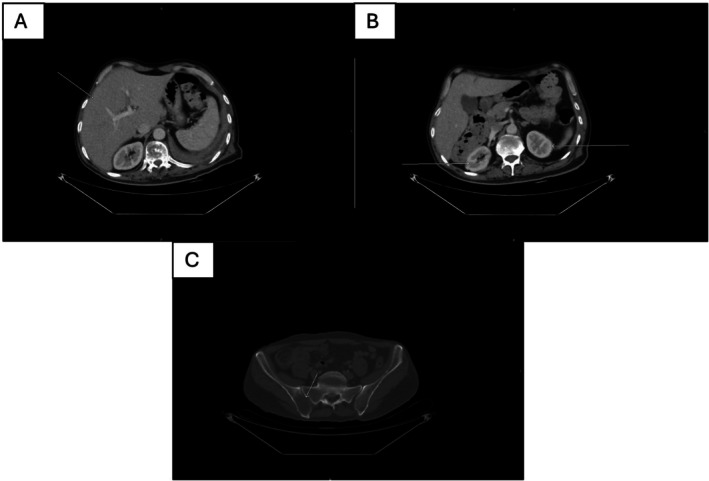
Contrast‐enhanced axial CT images demonstrating the absence of focal solid‐organ metastases and the presence of osseous involvement: (A) homogeneous hepatic parenchyma without focal lesions, (B) normal enhancement of both kidneys with no focal renal masses and (C) a lytic lesion involving the pelvic bone (arrow), consistent with metastatic disease.

A subsequent high‐resolution triphasic contrast‐enhanced CT scan of the abdomen demonstrated a liver of average size with homogeneous enhancement, patent portal and hepatic veins and no focal hepatic lesions. The spleen, pancreas, kidneys, adrenal glands, abdominal vasculature, and retroperitoneum were unremarkable, with no abdominal lymphadenopathy or masses identified. The previously noted lytic lesions involving the vertebrae and pelvic bones were again visualised, confirming metastatic involvement (Figure 4).

Histopathological examination of a tru‐cut biopsy specimen revealed a poorly differentiated adenocarcinoma with prominent signet ring cell differentiation. Immunohistochemical analysis showed diffuse positivity for cytokeratin 7 (CK7) and HepPar‐1, focal positivity for CDX2, and negativity for cytokeratin 20 (CK20), p63, thyroid transcription factor‐1 (TTF‐1) and D2‐40. Cytological examination of pleural effusion samples demonstrated inflammatory smears without malignant cells. Based on the morphological features and immunophenotypic profile, a diagnosis of hepatoid adenocarcinoma of the lung was established. Molecular profiling for actionable mutations (including EGFR and ALK rearrangements) and PD‐L1 expression analysis was not performed due to insufficient tissue sample.

Following confirmation of the diagnosis, the patient was initiated on systemic chemotherapy consisting of gemcitabine administered on days 1 and 8 combined with carboplatin on day 1, repeated every 3 weeks. He received a total of 3 cycles. Despite treatment, his clinical condition progressively deteriorated. The sequence of clinical events is summarised in Table 2.

**TABLE 2:** Clinical timeline of disease course.

Phase	Clinical events
Initial presentation	Persistent cough; initially diagnosed as pneumonia and treated with antibiotics outside our institution
Symptom progression	Progressive dyspnea, weight loss, fatigue, and diffuse bone pain
Oncology referral	Presentation to oncology clinic due to persistent and progressive symptoms
Diagnostic assessment	Laboratory studies and non‐contrast CT revealed consolidation, pleural effusion, mediastinal lymphadenopathy, and lytic bone lesions
Staging workup	Triphasic CT abdomen showed no focal hepatic lesion
Pathologic confirmation	Tru‐cut biopsy confirmed pulmonary hepatoid adenocarcinoma
Systemic therapy	Gemcitabine plus carboplatin initiated
Treatment course	Three cycles completed
Clinical deterioration	Progressive decline despite therapy

## 3 Discussion

Pulmonary hepatoid adenocarcinoma (HAL) is an exceptionally rare extrahepatic adenocarcinoma that recapitulates hepatocellular carcinoma–like morphology and expresses hepatocytic differentiation markers while arising in the lung. Reported literature consistently shows a predilection for middle‐aged/older men with heavy smoking exposure and an aggressive clinical course, with many patients already metastatic at diagnosis and overall survival generally poor. This case aligns with those hallmarks and adds an instructive ‘pneumonia mimic’ presentation with very rapid progression to widespread osseous disease [3, 4].

A major challenge in HAL is delayed recognition because early symptoms (cough, dyspnea, chest discomfort) and initial imaging can resemble infection or inflammatory lung disease. Recent reports describe HAL being interpreted as inflammation or mass‐like consolidation on CT, resulting in antibiotic therapy and postponed biopsy. In the present case, the patient was treated for pneumonia in initial presentation, yet symptoms persisted and evolved into dyspnea and systemic features (weight loss, fatigue), prompting oncologic evaluation approximately 2 months later. The thoracic CT demonstrated left upper‐lobe consolidation with pleural effusion and associated collapse, a pattern that can plausibly distract from malignancy—particularly if clinical reassessment is delayed when ‘pneumonia’ fails to resolve [6].

The extent of disease at staging underscores the biologic aggressiveness of HAL. This patient had mediastinal lymphadenopathy and multiple lytic lesions throughout vertebrae and pelvic bones, correlating with diffuse bone pain and functional decline. Advanced presentation is typical in pooled analyses and real‐world cohorts; bone and brain metastases are prominent metastatic sites, and nodal/liver involvement is also frequently reported. Of note, triphasic contrast CT showed no focal hepatic lesions, supporting a primary pulmonary origin rather than an occult hepatic primary with lung metastasis—an essential distinction because HAL can be morphologically indistinguishable from hepatocellular carcinoma (HCC) on routine histology [7].

Serum biomarkers should be interpreted with caution and not relied upon in isolation. Although hepatoid tumours are historically associated with alpha‐fetoprotein (AFP) production, AFP expression is variable in HAL, and cases without AFP elevation have been repeatedly documented [8]. Haninger et al. [9] proposed modified diagnostic criteria where AFP is not required if other markers of hepatic differentiation are present, emphasising that normal AFP does not exclude HAL. This point is directly illustrated here: AFP was normal despite fulminant metastatic disease, while CEA was markedly elevated, reminding clinicians that conventional ‘lung adenocarcinoma’ markers may be abnormal even when AFP is not.

Histopathology with a carefully constructed immunohistochemical (IHC) panel remains decisive for diagnosis and for resolving key differentials. The biopsy showed poorly differentiated adenocarcinoma with prominent signet ring cell differentiation. While signet ring morphology and focal CDX2 positivity can raise concern for a gastrointestinal primary, signet ring components have been documented within the spectrum of HAL [9]. In this context, the overall IHC profile is crucial: diffuse CK7 and HepPar‐1 positivity supports hepatoid differentiation in a carcinoma with pulmonary phenotype, while negativity for TTF‐1 and CK20 argues against typical primary pulmonary adenocarcinoma patterns on one hand and many colorectal primaries on the other (acknowledging that none of these markers are individually definitive). The absence of liver lesions on triphasic imaging, normal liver enzymes, and negative viral hepatitis screening further strengthens the interpretation of a primary pulmonary hepatoid tumour rather than metastatic HCC. Pleural fluid cytology was negative for malignant cells; however, this does not exclude malignant pleural involvement, as negative cytology may reflect sampling limitations or a predominantly inflammatory effusion in HAL [10].

Therapeutic decision‐making in HAL is difficult because no disease‐specific guideline exists; treatment is typically extrapolated from non–small cell lung cancer standards. Systematic reviews and updated narrative analyses suggest that complete surgical resection, when feasible in localised disease, offers the best chance for meaningful survival, whereas nodal/metastatic disease carries a poor prognosis with limited benefit from systemic therapy. In advanced HAL, platinum‐based chemotherapy has been used with inconsistent and often short‐lived responses. Emerging reports suggest that immunotherapy may have activity in selected cases, particularly when PD‐L1 expression is present, but evidence remains limited to small series/case reports. In our patient, gemcitabine plus carboplatin was administered for 3 cycles with continued clinical deterioration, reflecting the commonly reported chemoresistant, rapidly progressive course of metastatic HAL [11, 12].

Recent reports have provided insight into the molecular landscape of HAL. Isolated cases have demonstrated actionable alterations, including ALK rearrangements, with reported responses to targeted therapy, suggesting potential therapeutic relevance in selected patients [13]. In a small series, Pasricha et al. [14] evaluated molecular alterations alongside PD‐L1 expression and did not identify targetable mutations, although PD‐L1 positivity was observed in some tumours, indicating a potential role for immunotherapy. Moreover, Basse et al. [15] described a patient who responded to anti–PD‐L1 therapy despite negative PD‐L1 expression, highlighting that treatment response may not depend exclusively on PD‐L1 status. These findings underscore the importance of comprehensive molecular testing when feasible. In our case, mutation testing and PD‐L1 analysis were not performed due to insufficient tissue availability.

Overall, this case emphasises practical lessons for earlier recognition and accurate diagnosis of HAL. First, HAL should be considered in high‐risk patients (notably heavy smokers) with persistent consolidation or ‘pneumonia’ unresponsive to antibiotics—especially when constitutional symptoms and early metastatic features such as bone pain are present. Second, normal AFP should not reassure against HAL; diagnosis depends on integrating morphology with hepatocytic differentiation markers (e.g., HepPar‐1 and, when available, a broader hepatic panel) and excluding hepatic and gastrointestinal primaries through structured clinicoradiologic correlation. Finally, the fulminant trajectory in this patient underscores the need for rapid tissue diagnosis and multidisciplinary planning, because once disseminated, HAL outcomes remain poor despite standard NSCLC‐based systemic therapy.

In conclusion, this case highlights primary pulmonary hepatoid adenocarcinoma as a highly aggressive lung malignancy that can closely mimic nonresolving pneumonia and progress rapidly to widespread metastatic disease. Normal AFP levels should not delay suspicion or exclude the diagnosis; instead, timely biopsy with an appropriate immunohistochemical panel demonstrating hepatocytic differentiation and careful clinicoradiologic exclusion of a hepatic primary are essential for accurate classification. Given the poor response to conventional platinum‐based regimens in advanced disease, early recognition and prompt multidisciplinary evaluation are critical to optimise management, consider broader systemic options when available, and avoid diagnostic delay in patients with persistent consolidation and systemic symptoms.

## Author Contributions

All authors contributed to the diagnosis, management, and follow‐up of the patient. All authors participated in data collection, literature review, and manuscript preparation. All authors reviewed and approved the final version of the manuscript.

## Funding

The authors have nothing to report.

## Consent

The authors declare that written informed consent was obtained for the publication of this manuscript and accompanying images using the form provided by the Journal.

## Conflicts of Interest

The authors declare no conflicts of interest.

## Data Availability

Data sharing not applicable to this article as no datasets were generated or analysed during the current study.
